# Binding of *Plasmodium falciparum* to CD36 can be shielded by the glycocalyx

**DOI:** 10.1186/s12936-017-1844-6

**Published:** 2017-05-10

**Authors:** Casper Hempel, Christian William Wang, Jørgen Anders Lindholm Kurtzhals, Trine Staalsø

**Affiliations:** 1 Department of Clinical Microbiology, Centre for Medical Parasitology, Copenhagen University Hospital, Copenhagen, Denmark; 2 Department of Immunology and Microbiology, Faculty of Health and Medical Sciences, University of Copenhagen, Copenhagen, Denmark; 3 Department of Micro- and Nanotechnology, Technical University of Denmark, Kongens Lyngby, Denmark; 4 Department of Infectious Diseases, Rigshospitalet, Copenhagen, Denmark

**Keywords:** *Plasmodium falciparum*, Endothelial glycocalyx, Cytoadhesion, Malaria, *Var* genes, Azido sugars

## Abstract

**Background:**

*Plasmodium falciparum*-infected erythrocytes sequester in the microcirculation due to interaction between surface-expressed parasite proteins and endothelial receptors. Endothelial cells are covered in a carbohydrate-rich glycocalyx that shields against undesired leukocyte adhesion. It was investigated if the cellular glycocalyx affects the binding of *P. falciparum*-infected erythrocytes to CD36 in vitro.

**Methods:**

Glycocalyx growth was followed in vitro by using azido sugars and cationized ferritin detecting O-glycoproteins and negatively charged proteoglycans, respectively. *P. falciparum* (clone FCR3/IT) was selected on Chinese hamster ovary (CHO) cells transfected with human CD36. Cytoadhesion to CHO CD36 at 1–4 days after seeding was quantified by using a static binding assay.

**Results:**

The glycocalyx thickness of CHO cells increased during 4 days in culture as assessed by metabolic labelling of glycans with azido sugars and with electron microscopy studying the binding of cationized ferritin to cell surfaces. The functional importance of this process was addressed in binding assays by using CHO cells transfected with CD36. In parallel with the maturation of the glycocalyx, antibody-binding to CD36 was inhibited, despite stable expression of CD36. *P. falciparum* selected for CD36-binding recognized CD36 on CHO cells on the first day in culture, but the binding was lost after 2–4 days.

**Conclusion:**

The endothelial glycocalyx affects parasite cytoadhesion in vitro, an effect that has previously been ignored. The previously reported loss of glycocalyx during experimental malaria may play an important role in the pathogenesis of malaria complications by allowing the close interaction between infected erythrocytes and endothelial receptors.

## Background

Cytoadhesion of infected erythrocytes plays a key role in malaria pathogenesis and contributes to disease severity [1–5]. During the intra erythrocytic part of their life cycle *Plasmodium* spp. invade erythrocytes and remodel the erythrocytic surface both in terms of exposed proteins, nanoprotrusions (‘knobs’) and rigidity [6]. These changes render the infected erythrocytes susceptible to splenic removal and thus cytoadhesion to endothelial cells in the microcirculation is essential for parasite survival.

The cytoadhesion is mediated by variant surface antigens (VSA) that the parasites export to the erythrocyte surface [7]. The binding is a strong selective force in vivo and parasites have multiple VSAs binding to multiple ligands [8–10] including CD36, a well-known glycoprotein receptor [11]. Studies of cytoadhesion and its role in malaria pathogenesis have mostly been performed by various in vitro assays using recombinant proteins, glycans or immobilized cells as ligands [7, 10–13]. However, the cytoadhesion assays have so far ignored the endothelial glycocalyx, which is a thick, negatively-charged carbohydrate-rich matrix anchored to the cell membrane by proteins and lipids [14]. Although the glycocalyx has been studied extensively on endothelial cells it is commonly overlooked in malaria research despite its relevance for endothelial homeostasis [14, 15]. Previous studies indicate that malaria affects the endothelial glycocalyx thickness and structure [16]. The present study examined the effect that the glycocalyx may have on parasite cytoadhesion. It is well known that the endothelial glycocalyx shields leukocytes and platelets from undesired binding to the endothelium [17, 18]. This led to the proposal that cytoadhesion of parasite-infected erythrocytes may similarly be affected by the glycocalyx [19]. The glycocalyx grows continuously during in vitro culture [20] and in order to assess how this affected cytoadhesion a simple culture system was used to quantify changes in parasite binding to CD36 as a consequence of glycocalyx growth on Chinese hamster ovary (CHO) cells.

## Methods

### Cultivation of Chinese hamster ovary cells (CHO), endothelial cells and *Plasmodium falciparum* parasites

In short cultivation was performed essentially as previously described [12]. The following CHO cell lines were used: CHO K1 [CHO WT, Cat No CCL-61™, American Tissue Culture Collection (ATCC)] and CHO CD36 (stably express human CD36, Cat No CRL-2092™, ATCC). CHO cells were cultured in HEPES-buffered RPMI 1640 (Cat No 01-106-1A, Biological Industries) supplemented with fetal bovine serum (FBS, final concentration 10%, Cat No 10500064, Gibco, Thermo Fischer Scientific) and gentamicin (final concentration 50 µg/ml, Cat No 15710064, Gibco). Cells were grown at 37 °C at 5% CO_2_.

Immortalized, human cerebral microvascular endothelial cells (hCMEC/D3 [21]) were kindly provided by Pierre-Olivier Couraud (Institut Cochin, Paris, France). hCMEC/D3 cells were grown in ECM2 medium (Cat No CC-3156, Lonza) supplemented with growth factor bullet (Cat No CC-3202, Lonza). Cells were grown at 37 °C at 5% CO_2_. Passage 27–29 was used for the described studies.

*Plasmodium falciparum* strain IT/FCR3 was cultured in culture flasks at 37 °C, at 4% haematocrit in an atmosphere of 2% oxygen, 5.5% CO_2_ and 92.5% N_2_ [12]. They were grown in HEPES-buffered RPMI Cat No 01-106-1A, Biological Industries) supplemented with Albumax (final concentration 5 mg/ml, Cat No 11021029, Gibco), hypoxanthine (0.02 mg/ml, Cat No H9636, Sigma-Aldrich), l-glutamine (0.18 mg/ml, Cat No G5792, Sigma-Aldrich) and gentamicin (final concentration 50 µg/ml, Cat No 15710064, Gibco). Subculture with the addition of blood group O erythrocytes was done throughout the study. Human blood was obtained with verbal informed consent from healthy volunteers, a procedure that is permitted without ethical approval from the Ethics Committee in the Capital Region of Denmark.

### Seeding cells at different densities

Several seeding densities were tested in order to obtain a confluent monolayer at the time of the experiment. For CHO cells the following densities were used: confluent day 1: 8 × 10^4^ cells/ml, confluent day 2: 2.5 × 10^4^ cells/ml, and confluent day 4: 6 × 10^3^ cells/ml. For endothelial hCMEC/D3 cells the following densities were used: confluent day 1: 2 × 10^5^ cells/ml, confluent day 2: 10^5^ cells/ml, and confluent day 4: 5 × 10^4^ cells/ml. These densities were seeded in 24- and 96-well plates and in transwell inserts for the experiments described below.

### Live labelling of extracellular glycosylation

CHO and hCMEC/D3 cells were seeded in chamber slides (Ibidi, Germany) and extracellular carbohydrates detected by adding *N*-azidoacetylgalactosamine-tetraacylated (GalNaz, 50 µM, Cat No 88905, Thermo Fischer Scientific) to the culture medium. After a various number of days in culture the medium was removed and cells washed with 2% FBS in phosphate buffer (0.15 M Sørensen’s Buffer). The azido sugars were detected by incubating the cells with 100 mM DyLight488-labeled phosphine for 60 min at 37 °C (Cat No 88907, Thermo Fischer Scientific) diluted in phosphate buffer. Negative controls were cells not fed GalNaz but only the normal cell culture medium. Cells were co-stained with Hoechst 33342 (5 µg/ml, Cat No H3570, Thermo Fischer Scientific) and cell mask orange (1000× dilution, Cat No C10045, Thermo Fischer Scientific). After 60 min incubation cells were washed with FBS supplemented phosphate buffer. Labelling was assessed in live cells with a confocal microscope (Zeiss LSM780) at 37 °C using a 20× objective (NA 0.8).

### Analyses of azido sugar binding

All cells were imaged with identical microscopy settings using Carl Zeiss software (Zen). Image analyses were performed using Image J version 1.47 [22]. The green channel was separated from the original image and a transect was made from the upper left corner to the lower right corner. The raw data are shown as a histogram of intensity. Imaging depth was 16 bit and the maximal intensity is 65,355.

### Transmission electron microscopy (TEM)

Cells were grown on hanging polyethylene terephthalate filters with pore size 0.4 µm (Cat No PIHT12R48, Milipore, Merck, Germany) and seeded in different densities allowing them to become a confluent monolayer at days 1, 2 and 4. When confluent, cells were washed in 5% bovine serum albumin (BSA, Cat No A8022, Sigma Aldrich) and incubated with 0.5 mg/ml cationized ferritin (Cat No F7879, Sigma Aldrich) in 5% BSA for 30 min at room temperature. Cells were initially washed in 5% BSA then in 0.2 M cacodylate buffer [Cat No 11653, electron microscopy sciences (EMS)]. Cells were fixed ON in 2.5% glutaraldehyde (Cat No 16210, EMS) in 0.05 M cacodylate buffer and dehydrated according to standard methods [23]. Cells were osmificated at 1% for 60 min. (Cat No AGR1017, Agar Scientific) and infiltrated with an epoxy resin (Cat No T031, Embed 812 Medium, TAAB) by using propylene oxide as an intermediate (Cat No 20401, EMS). Cells were initially cut as semi thin sections at 1 µm and stained with toluidine blue. Then cut for TEM at 50 nm thickness and stained with uranyl acetate and lead citrate (EMS) as previously described [23]. All TEM was performed by using a Philips CM100 equipped with an Olympus Veleta camera connected to a workstation with SIS Analysis software (iTEM).

### Selection procedures

Parasites were selected for binding to CHO CD36 cells as described in detail previously [12]. The selection procedure was repeated at least four times. All CHO CD36 cells were seeded and used for selection the following day.

### Binding experiments

In essence binding experiments were performed as described in detail previously [12]. All binding experiments were performed using 1% haematocrit and 20% parasitaemia. Binding to CHO CD36 was compared with binding to CHO WT cells and with unselected IT/FCR3 parasites and uninfected, healthy erythrocytes. Inhibition of binding to CD36 was performed as described previously [12] where CD36 antibody at different concentration was incubated with CHO CD36 cells and allowed to bind prior to the addition of parasites.

### CD36 expression

The expression of CD36 in CHO cells was compared with an on-cell ELISA design. After growth a different number of days, the cells were washed in PBS and fixed with formaldehyde 1% for 5 min. After washing, cells were blocked with 5% BSA for 30 min at room temperature and incubated for 60 min at room temperature with primary antibodies at 1 µg/ml (5% BSA in PBS as diluent). The primary antibody was CD36 (clone FA6.152, Cat No IM0765, Beckman Coulter). Cells were washed with PBS and bound antibodies detected by horse-radish peroxidase-conjugated anti-mouse antibodies (Cat No P0447, Dako). Labelling was quantified with a luminescent substrate (Cat No 34094, West femto, Thermo Fisher Scientific) in a luminescence reader (500 ms exposure. Victor2, Perkin Elmer, USA). After detection, substrate was removed and cells lysed with 50 µl triton x-100 (0.1% in PBS, Sigma-Aldrich). Protein content in the lysate was determined by a modified Lowry method (DC kit, Bio Rad, USA) and read at 650 nm (Multiscan, Thermo Fischer).

To test for total CD36 expression CHO cells grown for 1 and 4 days were lysed in ice-cold RIPA buffer (Cat No R0278, Sigma-Aldrich) with protease inhibitors (Cat No, 04693124001, Complete Mini, Roche). Lysate was spun and the supernatant was collected. Protein content was determined with Lowry method (Cat No 5000112, DC protein assay kit, Bio-Rad). 2 µl (5 µg protein/ml) was spotted on nitrocellulose membrane (Cat No 1620150, Bio-Rad), left to dry and blocked with 5% skim milk powder in tris-buffered saline (Sigma-Aldrich) for 60 min. The membranes were incubated over night with primary antibodies as described above and detected with matching Alexa647-conjugated secondary antibodies. An antibody against β-tubulin was used as a loading control (1000× diluted, Cat No Ab6046, Abcam). Content was quantified by using a LAS4000 scanner (CY5 filter, GE Healthcare) and analysed by densitometry using Image J 22.

### *Var* gene profiling

The expression profile of *var* genes was performed by quantitative PCR analyses of mRNA. Ring-stage parasites were enriched with sorbitol as previously described [24]. The washed pellet (100 µl) was thoroughly mixed with 900 µl Trizol (Cat No 15596026, Thermo Fischer Scientific) and stored at −80 °C use. RNA was reverse transcribed from random hexamers, using Superscript II (Cat No 18064014, Thermo Fischer Scientific), according to the manufacturer’s instructions. Quantitative primers for each *var* gene of the *P. falciparum* clone IT/FCR3 and quantitative PCR was performed on a Rotorgene RG-3000 thermal cycler (Corbett Research) was as previously described [25], where gene-specific standard curves were produced by determining the amplification efficiency relative to the single copy housekeeping gene, *seryl*-*tRNA synthetase*, based on quantitative measurements of 10-fold dilutions of genomic DNA and used to calculate the transcript copy number of each gene in tested cDNA.

### Statistical analyses

Data from binding experiments were assessed for equal variance and Gaussian distribution by using R [26]. Data followed the criteria and were analysed with one-way ANOVA followed by post hoc tests (Holm–Sidak correction). Data from on-cell ELISA failed equal variance tests and were analysed with non-parametric tests (Kruskal–Wallis followed by Mann–Whitney U test). If data followed criteria for parametric testing they are shown as mean with standard deviation, otherwise as medians with interquartile ranges. All experiments were run in quadruplicate and repeated at least twice or in triplicate and repeated at least thrice. Graphs are designed in Graphpad Prism (version 7).

## Results

### Cells used for studies of parasite adhesion increase their glycosylation as a function of time in culture

**Fig. 1:**
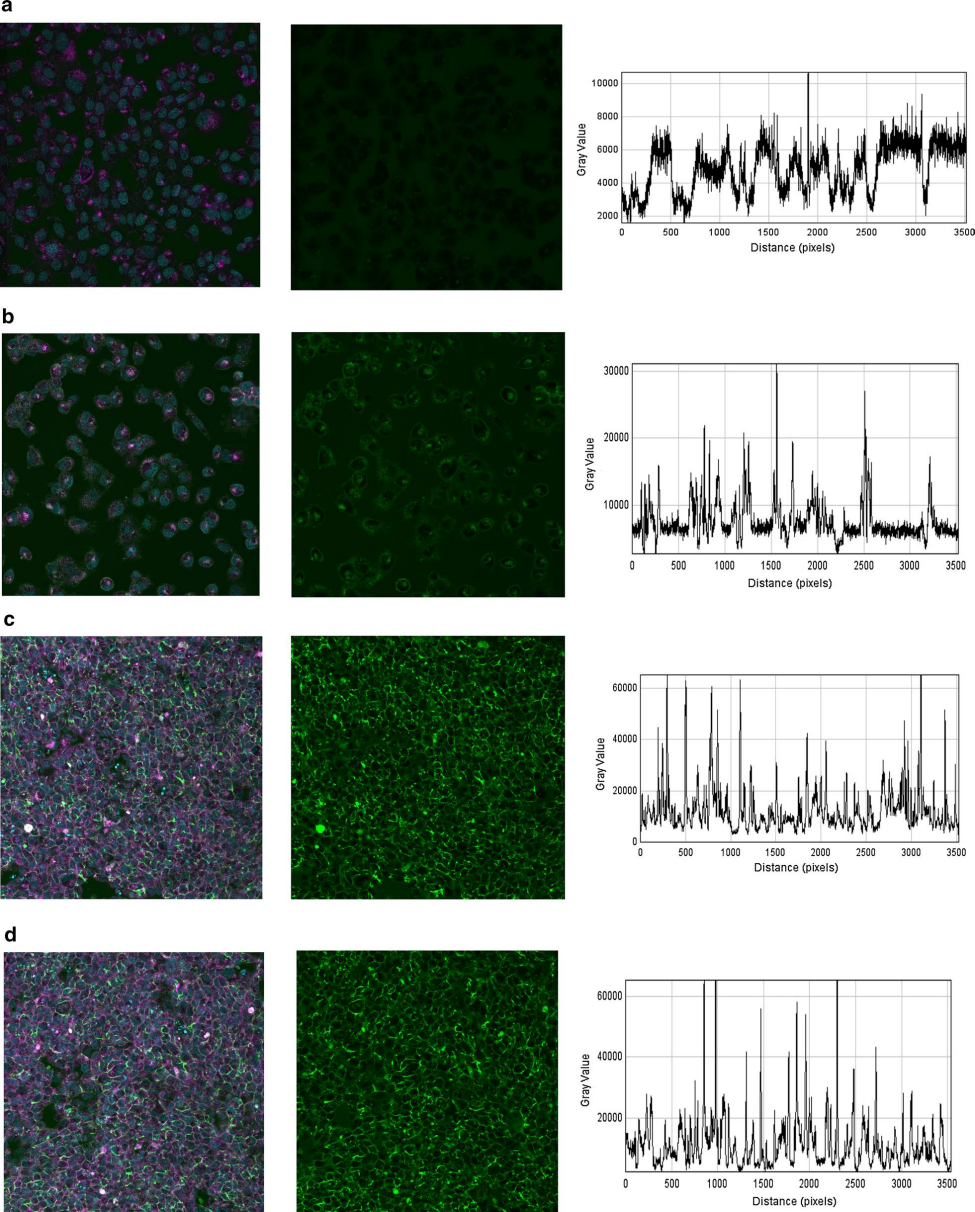
Azido sugars are taken up by CHO cells and presented in increasing amount as a function of time grown with sugars. **a** After 1 day only limited amount of azido sugar is detected on the cell surface of CHO WT cells. The *left* panel shows cell labelled with cell mask orange (*magenta*), nuclei (*cyan*) and azido sugar (*green*). The *middle* panel is the signal from the green channel while the *right panel* is a transect from the *upper left corner* to the *lower right* in terms of intensity in the green channel. The signal is indistinguishable from background levels. **b** The three panels show the same information as in **a** but at day 2 after adding azido sugars. At day 2 notable amounts are present. **c** At day 4 after adding azido sugars a significant amount is detected on almost all cells. **d** Similarly, in CHO CD36 cells azido sugars are externalized to a high level after 4 days in culture. The degree of externalization is comparable in CHO CD36 and CHO WT cells. All cells are imaged live with a 20× objective

Inspired by [20] the study aimed at investigating how the glycocalyx changes on CHO cells, commonly used for studies of cytoadhesion [12]. When azido sugars are added to the culture medium the cells start incorporating these into O-linked glycoproteins normally expressed on the cell surface [27] and this can be used to visualize the glycocalyx. When CHO cells were stripped of surface proteins by trypsinization and then grown for increasing time in culture in the presence of azido sugars the daily increase in surface expression of the glycocalyx became evident (Fig. 1). After 1 day of co-culture negligible amounts were detected on the intact cell surface (Fig. 1a). After 2 days surface staining of the glycocalyx was markedly increased and the thickness was further increased after 4 days in co-culture (Fig. 1b, c). Azido sugars did not affect cell growth or cell viability. Then CHO cells transfected to express human CD36; a common endothelial receptor targeted by the parasite for cytoadhesion [10] were used. Similar effects on the growth of the glycocalyx were noted for CHO CD36 cells when cultured in the presence of azido sugars (Fig. 1d).

**Fig. 2:**
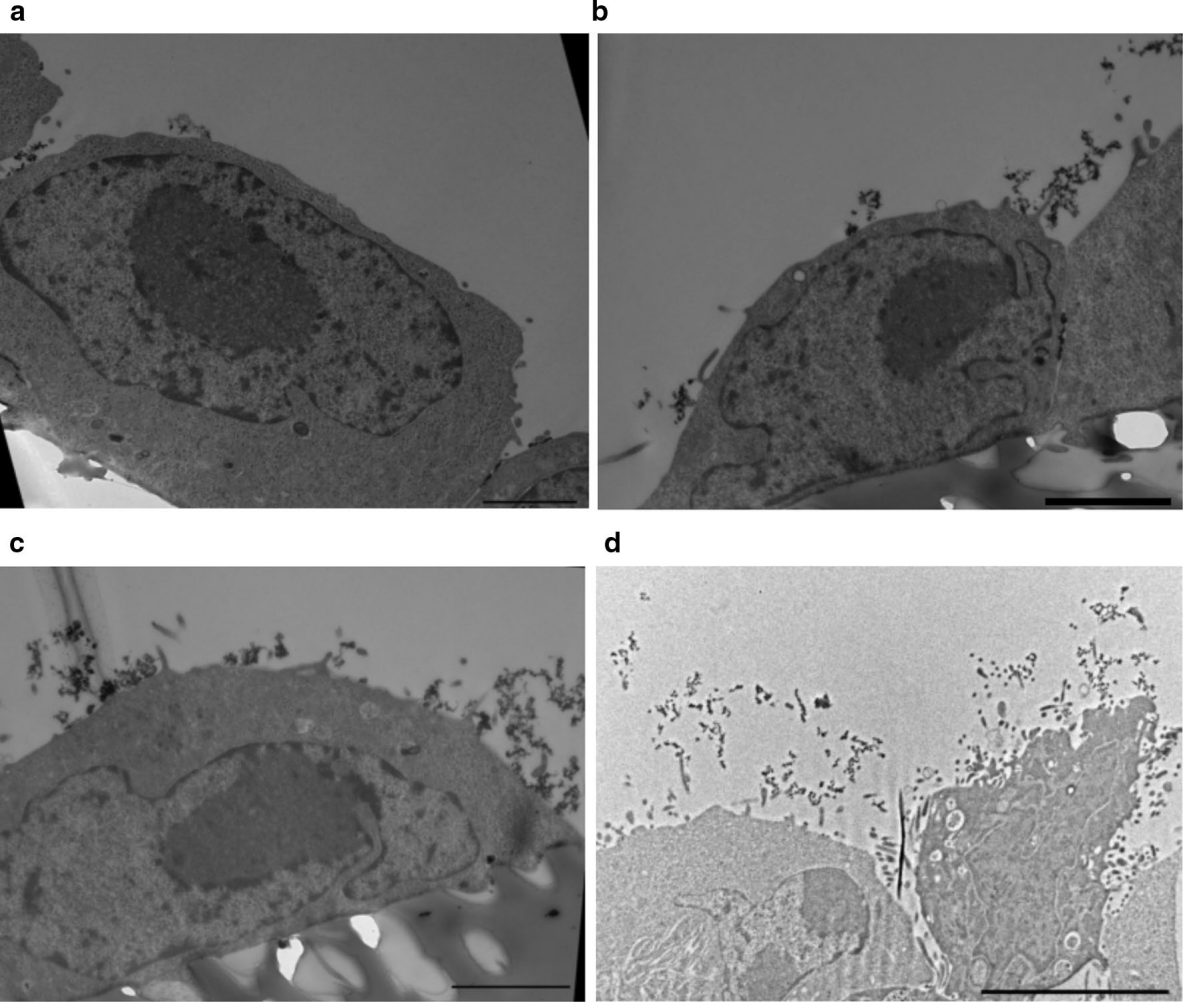
Staining the glycocalyx of CHO WT cells with cationized ferritin shows increasing amounts after several days in culture. Ferritin is seen as *spherical dark dots* (11 nm in diameter). **a** One day after seeding CHO WT cells have limited glycocalyx. Most ferritin labels glycans in the intercellular clefts. However, also sparse labelling on the cell surface was noticed. **b** Similar to **a** glycans in the intercellular clefts are labelled but after 2 days in culture multiple clusters of ferritin are evident on the cell surface. **c** After 4 days the cell surface is covered by ferritin staining. **d** CHO CD36 cells at day 4 after seeding. As for CHO WT cells the glycocalyx covers the entire cell surface. *Scale bars* show 2 µm in **a**, **b**, **c** and 5 µm in **d**

Apart from the O-linked glycoproteins [28], visualized by azido sugars, the glycocalyx also consists of proteoglycans with long glycosaminoglycan side chains [14]. These can be labelled with cationized ferritin and visualized by electron microscopy [29]. Similar to azido sugar labelling, an increased glycocalyx coverage of cells with time was found (Fig. 2). One day after seeding ferritin mostly labelled the confluent monolayer at the intercellular junctions (Fig. 2a). As noticed with azido sugar labelling, glycocalyx covering of CHO cells was considerably more prominent at day 2 and 4 (Fig. 2b, c). This was also seen for CHO CD36 (Fig. 2d).

### Days in culture affect parasite recognition of the cell surface

**Fig. 3:**
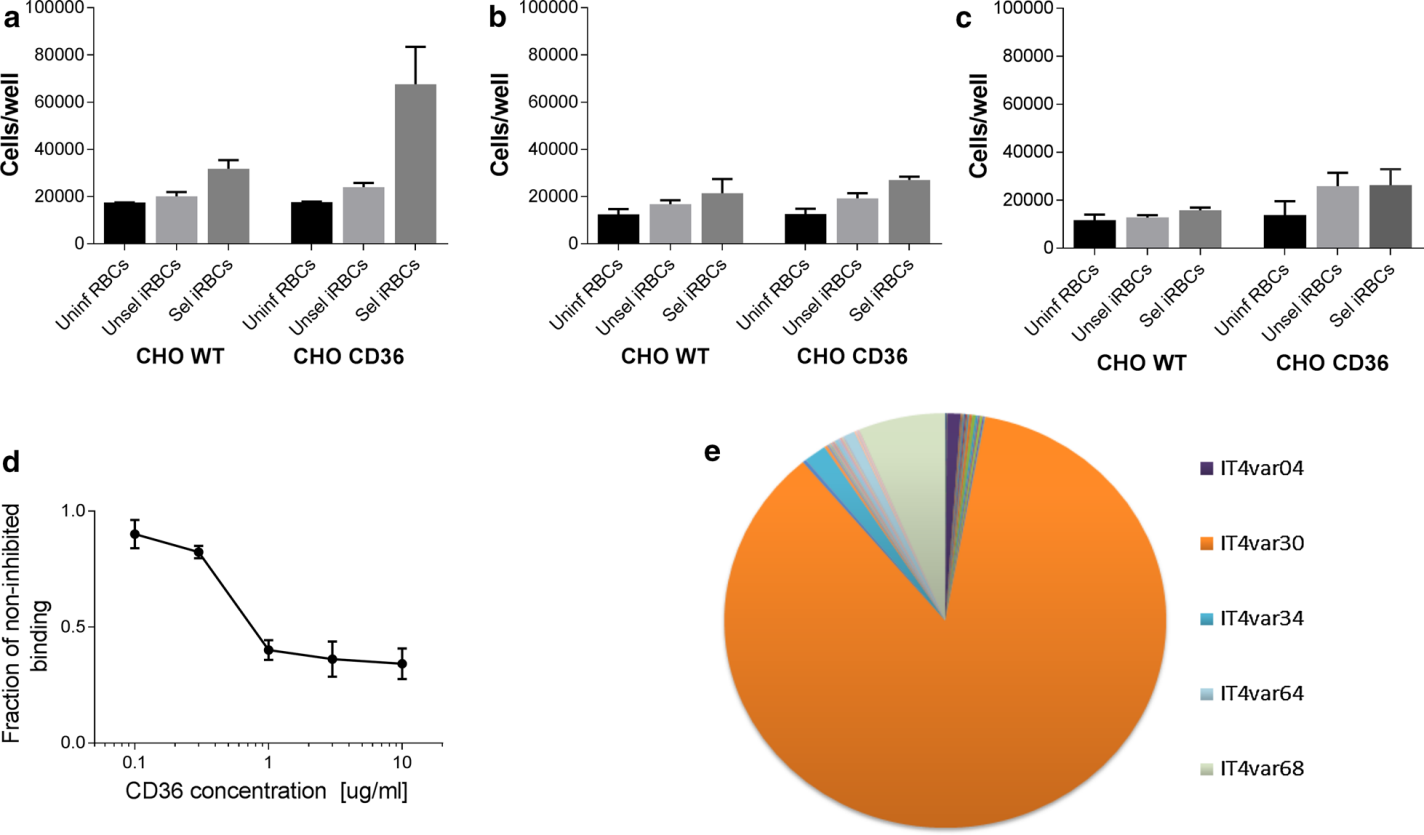
Parasites selected for CHO CD36 binding show reduced binding when cells have been in culture for multiple days. **a** After 1 day in culture CD36-selected parasites bind significantly better to CHO CD36 cells compared with unselected and uninfected erythrocytes (p < 0.0001). CD36 selected parasites also bind CHO WT cells better than unselected parasites but without being statistically significant (p > 0.05). **b** In contrast after 2 days in culture no specific binding was noticed when selected vs unselected parasites were compared (p > 0.05). However, selected parasites bound significantly better to both CHO WT and CHO CD36 compared with uninfected erythrocytes (p < 0.03). **c** After 4 days in culture no statistical difference in cytoadhesion was observed (p > 0.08). **d** Specificity of CD36 binding was assessed by inhibiting CD36 with an antibody directed against the receptor. At the highest antibody concentration applied binding was inhibited to approximately 30% of the initial binding. **e** To assess whether the phenotype was of a type that has been predicted to bind CD36 *var* gene profiling was performed. The five most abundant transcripts are shown. *Bar graphs* in **a**–**c** represent mean values and *error bars* show standard deviation. The *line graph* in **d** shows mean values with standard deviations

Then the functional effect of increased coverage with glycans over time was assessed by quantifying binding of *P. falciparum* selected for cytoadhesion to CD36. Unselected parasites bind poorly to CHO cells regardless of CD36 expression (Fig. 3a). After pre-selecting parasites to CD36 they showed specific recognition of CD36 and recognized CD36-transfected CHO cells significantly better compared with WT CHO cells and unselected parasites (Fig. 3a, p < 0.0001).

CD36-selected parasites also bound to CHO WT but no significant difference was found compared to unselected parasites (Fig. 3a, p > 0.05). This pattern was repeatedly observed when CHO cells were trypsinized and seeded the day before the experiment. Interestingly, after 2 days in culture the specific recognition of CD36 disappeared (Fig. 3b, p > 0.05), yet, the selected parasites were bound in significantly higher numbers in both cell lines compared with uninfected erythrocytes (p < 0.03). The same loss of specific CD36 recognition was noticed after 4 days in culture (Fig. 3c, p > 0.07).

To assess the specificity of the binding, CD36-expressing CHO cells were seeded for 1 day and then CD36 antibodies were added to block parasite binding sites. By doing so the initial binding was dose-dependently reduced to approximately 30% (Fig. 3d). Quantitative-PCR was performed to assess the *var* gene transcript profile of parasites selected for CD36 binding used in the binding assays. The most prominent *var* transcript was IT4var30 comprising approximately 85% of the total *var* transcripts (Fig. 3e). The encoded IT4var30 PfEMP1 protein contains a cysteine-rich interdomain region-α2 (CIDRα2) domain predicted to bind CD36 [30].

### Time in culture does not change total amount of CD36 expression but modifies antibody accessibility to CD36

**Fig. 4:**
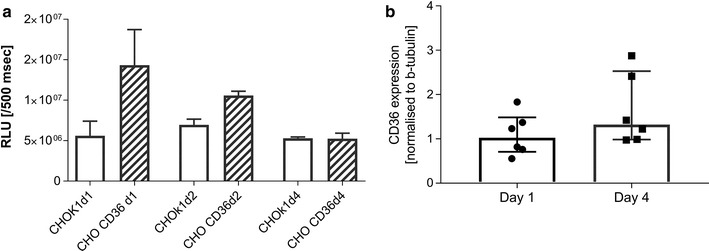
Antibody recognition of CD36 is also reduced as a function of time in culture. **a** Day 1 after seeding significantly higher levels of CD36 was detected when compared to WT cells without CD36 (p = 0.03). This difference was not observed 2 and 4 days after seeding (p > 0.2). Data are expressed as relative light units (RLU) divided by total protein content. **b** The total level of CD36 was not changed as a function of time (p = 0.2). *Bar plots* show median values and interquartile ranges

To directly demonstrate the reduction of CD36-binding, an on-cell ELISA using monoclonal anti-CD36 was used [31]. Antibody recognition of CD36 was significantly reducted as the CHO CD36 cells aged (Fig. 4a). At day 1 significantly higher levels of CD36 were detected on CHO CD36 cells compared with WT cells (p = 0.03, Fig. 4a). At day 2 CD36 detection was still higher, although not significantly, in CHO CD36 cells, and at day 4 post seeding the difference between cell types had disappeared (p > 0.2, Fig. 4a).

One explanation for the differential binding as a function of time could be a loss of CD36 when cells were grown for multiple days. To assess the total quantity of CD36, CHO cells were grown for increasing time, lysed and analysed for CD36-expression by dot blot. The data were normalized to β-tubulin [32]. This experiment showed no change in total CD36 expression over the 4-day-period of the experiment (p = 0.2, Fig. 4b). As expected no human CD36 was detected in WT CHO cells.

### Human brain microvascular endothelial cells show comparable modification of cell surface glycosylation when cultured for multiple days

**Fig. 5:**
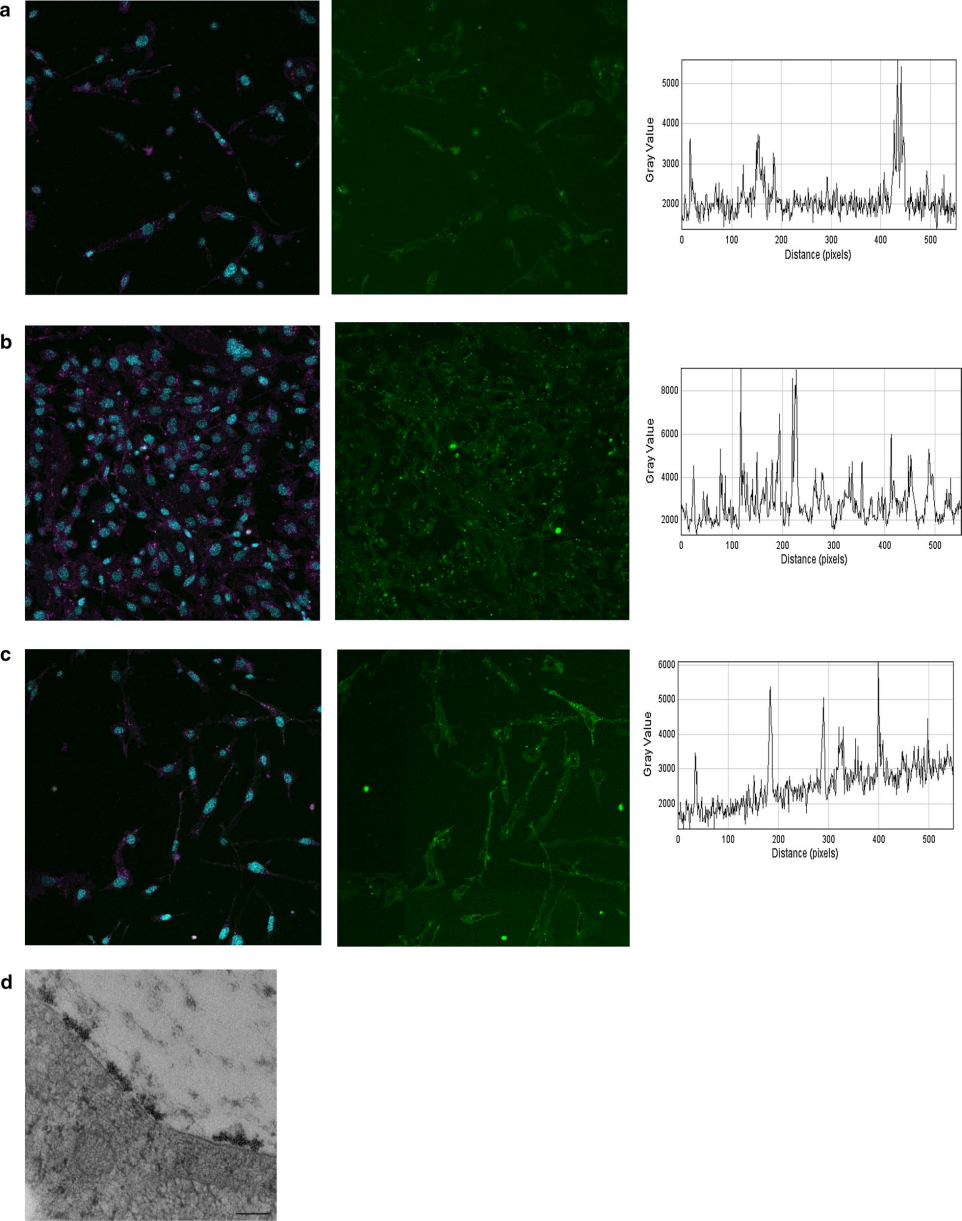
hCMEC/D3 cells also shows spatio-temporal development of the glycocalyx Endothelial cells were fed azido sugars for various days and externalization was quantified by light microscopy. **a** After 1 day in culture only limited staining is seen. The *left panel* shows cell labelled with cell mask orange (*magenta*), nuclei (*cyan*) and azido sugar (*green*). The *middle panel* is the signal from the green channel while the *right panel* is a transect from the *upper left corner* to the *lower right* in terms of intensity in the green channel. **b** At day 2 after seeding more staining is noticed although staining mainly is identified as local peaks. **c** After 4 days the staining is comparable with day 2. **d** Cells were also processed for electron microscopy. At day 4 several tufts are seen on the endothelial surface. *Scale bar* in **d** corresponds to 300 nm

To evaluate if these factors only apply to CHO cells, human brain microvascular endothelial cells were grown for various days to compare spatio-temporal development of the glycocalyx. Similar to what was observed with CHO cells, an increase in glycocalyx intensity over time was noticed although not as pronounced (Fig. 5a–c). This was confirmed by electron microscopy where mostly focal tufts of ferritin were noted (Fig. 5d).

## Discussion

Malaria parasite cytoadhesion has been widely studied since it is believed to be involved in pathogenesis and malaria severity [11]. So far, in vivo studies of the cytoadhesion of *P. falciparum*-infected erythrocytes have been difficult due to the lack of a good animal model [33], and studies of malaria-induced changes to the microcirculation in vivo rely mostly on ophthalmoscopy [34]. Thus, in vitro models have been used for most in-depth studies of cytoadhesion [10, 12, 35, 36].

The present data show that in vitro cytoadhesion to CD36 could be blocked by glycocalyx growth in CHO cells. CD36 selected parasites recognized CD36 on CHO cells but only when the cells were not covered by a glycocalyx. In this case specific recognition was reduced significantly.

Two independent markers of glycocalyx growth were used: azido sugars visualizing total O-linked proteins and cationized ferritin binding, which mainly reacts with negatively charged glycosaminoglycan chains on proteoglycans [15]. This allowed for confirmation of the previously described spatio-temporal in vitro growth of the glycocalyx in cell cultures [20]. This suggests that the thickness of the glycocalyx also has functional importance in terms of cytoadhesion in malaria and that it should be taken into account.

There is a significant difference in the two described labelling approaches for the glycocalyx. By using the azido sugars one can quantify the processing of glycocalyx by the cells. Thus, if the cells do not export O-linked glycoproteins to the cell surface no labelling will be observed. The method does not demonstrate O-linked glycoproteins that had been formed prior to adding GalNaz. In contrast, ferritin labelling will detect all negative surface charge present when added. Demonstration of ferritin shortly after trypsinization gives indications of either a robust negatively charged surface coat that is resistant to trypsin, or a coat that is very quickly rebuilt. Together the two approaches give a picture of the dynamics taking place at the cell surface confirming the cell surface changes over time as previously reported [20]. Thus, after trypsinization the majority of the negatively charged glycans are located *intracellularly* and they require 2–4 days in culture to reach an extent, where they are able to cover surface receptors such as CD36.

An alternative explanation of these findings could be a reduced CD36 expression on the CHO cells with time in culture. However, dot blot analysis showed that CD36 expression was stable during 4 days of incubation. In contrast, antibody accessibility and binding to CD36 was reduced at the same times that parasite binding was inhibited. This further supports a role of the increased glycocalyx thickness in inhibiting binding.

Although the parasite cell line used was selected for CD36 binding a complete inhibition of binding with anti-CD36 was not possible. In concordance with this some degree of unspecific binding by unselected parasites was noticed. Interestingly, anti-CD36 reduced the binding to levels comparable to background binding to WT CHO cells.

The profile of the CHO CD36 selected parasite isolate matches what has previously been shown [30]. *Var* genes encode more than 60 types of the protein called *P. falciparum* erythrocyte membrane protein-1 (PfEMP1), which is composed by multiple CIDR and Duffy binding-like (DBL) domains [37]. *P. falciparum* exports multiple VSAs to the cells surface and in this study it was not assessed whether one or more of these smaller VSA contribute to CD36 adhesion.

Various PfEMP1 variants have been shown to exhibit binding to both glycans and proteins [11, 13, 30, 36]. It would be obvious to take into account how the glycocalyx [14] affects binding to ligands involved in malaria pathogenesis. A loss of glycocalyx has been demonstrated in murine models of malaria [16] and the inflammatory conditions may prime for both loss of glycocalyx as well as upregulation of adhesion molecules including CD54 enabling improved cytoadhesion [19].

The glycocalyx is a permeability barrier and plays a considerable role in preventing loss of albumin via the kidney [38]. In vivo imaging has also revealed that different sizes of dextran have different permeability through the glycocalyx. A 70 kDa fluorescein dextran only enters the glycocalyx after enzymatic removal of glycans [39] and these molecules are half the molecular weight of an IgG molecule. The glycocalyx could be expected to block binding to large molecules such as immunoglobulins as well as infected erythrocytes in vivo.

From a glycocalyx perspective, the use of static binding assays may be questioned due to the tendency of erythrocytes to sink into the glycocalyx if they are placed on top of it [40]. Reduced erythrocyte binding may not only be caused by steric hindrance but also by a different surface charge of the immobilized cells. The use of endothelial cell lines for in vitro studies of parasite adhesion could also be reconsidered since such cell lines appeared to develop a less pronounced glycocalyx layer in vitro compared with what has been found in vivo [41, 42]. In accordance with previous studies less intense staining of glycocalyx on brain endothelial cells compared with CHO cells was also noticed in this study. Thus, the functional importance of the glycocalyx in endothelial cells was not tested.

The present data suggest that the loss of endothelial glycocalyx during malaria may be an important factor allowing the sequestration of infected erythrocytes to endothelial receptors. Although the study only shows data for CD36, further studies should address the possibility of a similar effect on receptors implicated in cerebral malaria such as CD54 and endothelial protein C receptor. It is clear that the inflammatory conditions during clinical malaria may be a direct cause of the shedding of the glycocalyx [43]. In line with this, glycocalyx loss in murine malaria models has been shown; the loss being more pronounced in severe than in uncomplicated disease [16]. However, the glycocalyx loss was only significant several days after parasites were detected in the bloodstream, and there is a need to study the initial steps allowing contact between infected erythrocytes and surface receptors. Conversely, it was recently speculated that the glycocalyx is essential for the propagation of malaria since the parasites rely heavily on cytoadhesion in order to avoid splenic destruction [19]. Glycan-mediated cytoadhesion has been known for several years [13, 44, 45] but an understanding of the spatial and temporal interplay between parasite VSA, glycocalyx and endothelial protein receptors is lacking.

## Conclusion

This paper points towards careful evaluation of conclusions drawn from in vitro cytoadhesion studies. Specifically, there is a need to evaluate the role of the glycocalyx in cytoadhesion studies, both as a factor that can influence protein–protein binding to glycoproteins negatively as seen in this study but also as a potential co-receptor for protein–protein interactions. These aspects of cytoadhesion could be evaluated by using specific glycosidases removing carbohydrate components from target proteins or by using cells with genetically modified glycans [46].

## Abbreviations

CHO: Chinese hamster ovary
CIDR: cysteine-rich interdomain regions
DBL: duffy binding-like
PfEMP1: *P. falciparum* erythrocyte membrane protein-1
VSA: variant surface antigen
WT: wild type
